# The Role of Magnesium in Parkinson’s Disease: Status Quo and Implications for Future Research

**DOI:** 10.3390/ijms25158425

**Published:** 2024-08-01

**Authors:** Michal Cibulka, Maria Brodnanova, Erika Halasova, Egon Kurca, Martin Kolisek, Milan Grofik

**Affiliations:** 1 Biomedical Centre Martin, Jessenius Faculty of Medicine in Martin, Comenius University in Bratislava, 036 01 Martin, Slovakia; michal.cibulka@uniba.sk (M.C.); maria.brodnanova@uniba.sk (M.B.); erika.halasova@uniba.sk (E.H.); 2 Clinic of Neurology, Jessenius Faculty of Medicine in Martin, Comenius University in Bratislava, 036 01 Martin, Slovakia; egonkurca@gmail.com

**Keywords:** magnesium homeostasis, Parkinson’s disease, neurodegeneration, pathomechanisms

## Abstract

Neurodegenerative diseases represent an increasing economic, social, and, above all, medical burden worldwide. The second most prevalent disease in this category is Parkinson’s disease, surpassed only by Alzheimer’s. It is a treatable but still incurable systemic disease with a pathogenesis that has not yet been elucidated. Several theories are currently being developed to explain the causes and progression of Parkinson’s disease. Magnesium is one of the essential macronutrients and is absolutely necessary for life as we know it. The magnesium cation performs several important functions in the cell in the context of energetic metabolism, substrate metabolism, cell signalling, and the regulation of the homeostasis of other ions. Several of these cellular processes have been simultaneously described as being disrupted in the development and progression of Parkinson’s disease. The relationship between magnesium homeostasis and the pathogenesis of Parkinson’s disease has received little scientific attention to date. The aim of this review is to summarise and critically evaluate the current state of knowledge on the possible role of magnesium in the pathogenesis of Parkinson’s disease and to outline possible future directions for research in this area.

## 1. Introduction

Neurodegenerative disorders represent a heterogeneous group of maladies, significantly contributing to the morbidity and mortality of the global population. Of these, Parkinson’s disease (PD) and Alzheimer’s disease are the most prevalent. Epidemiological studies show an alarming increase in the prevalence of PD in the western population. Data from 2016 indicate that 6.1 million people had PD globally, representing more than the double amount compared to 2.5 million cases in 1990. Moreover, it is estimated that its prevalence will grow rapidly (12 million cases globally anticipated in 2040) in the future as a result of the ageing of the population as well as the lifestyle preferences of the Western population [1]. The exponential growth of prevalence, together with some other characteristics, leads some experts in the field to refer to PD as a Parkinson pandemic. At the same time, PD-related healthcare costs are significant. Only in the United States, the total economic burden of PD was USD 51.9 billion in 2017 [2].

Most of the research (and funding) in the PD field is focused on treatment strategies. As a result, numerous new molecules as well as electrophysiological approaches have been introduced [3]. Nevertheless, the prevalence of PD is still growing, and none of the available treatment approaches lead to the restoration of patients’ motor functions and/or halt the progression of the disease. It is thus more than relevant to focus more on prevention, which is a very promising approach, taking into account that genetics is a minor risk factor in sporadic form [4], which is in turn responsible for approximately 90% of PD cases. The rest of the risk as well as protective factors are usually hidden behind the terms ‘’environment“ and ‘’lifestyle“ [5]. Among the strongest risk factors are pesticides’ use, exposure to organic solvents and metals, air pollution, physical inactivity, and head injury. The most important protective factors include tobacco and caffeine intake, physical activity, and a healthy diet rich in grains, vegetables, and fruit. Identifying risk factors and promoting protective factors (tobacco and caffeine) after deeper analysis of the mechanism of action should be the cornerstone of a personalised, preventive, and protective approach.

**Figure 1:**
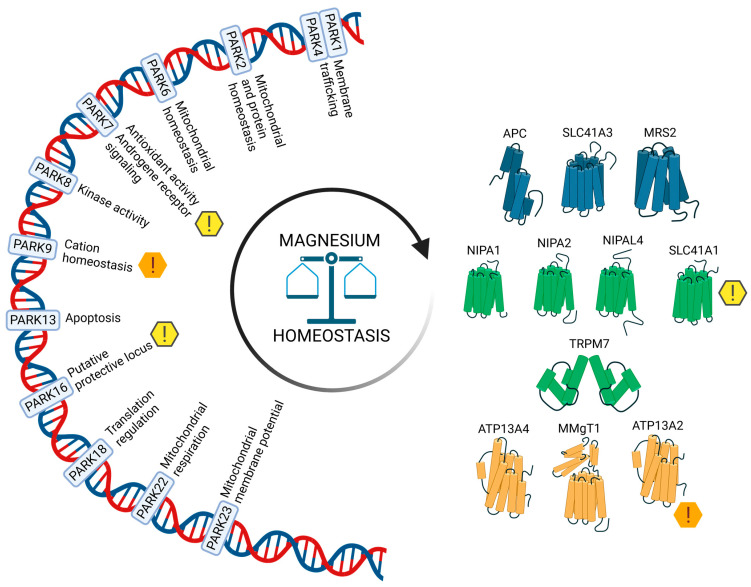
Magnesium transporters in the CNS- and Parkinson’s disease-related genetic loci relevant for magnesium homeostasis. The gene encoding for the putative magnesium transporter ATP13A2 is located in the PARK9 *locus*, and the gene encoding for the sodium–magnesium exchanger *SLC41A1* is a member of the *PARK16 locus*, together with *SLC45A3*, *NUCKS1*, *RAB7L1*, and *PM20D1*. Other *loci* encode for proteins, involved in a plethora of cellular processes, that directly modulate, or are influenced by, magnesium status. Cell membrane Mg transport systems are depicted in green, mitochondrial Mg transport systems in blue, and organelle Mg transport systems in yellow.

Magnesium (Mg) is an essential dietary factor. Growing data from both animal and human studies over the past ten years support the assumption that magnesium deficiency may be one of the major contributors to the pathophysiology of sporadic PD. The development of typical symptoms and neurodegeneration of specific brain areas are the two most noticeable pathophysiological hallmarks of PD. In the case of PD, a pathogenic mechanism that is still only poorly understood affects dopaminergic neurons in the pars compacta region of the substantia nigra (SNPC). Clinical signs of dopaminergic neurons’ loss include tremor, stiffness, and other motor and/or non-motor symptoms. There are two pathophysiologically distinct types of PD: the familial form and the sporadic form. Familial PD cases develop as a result of pathogenic variants in the genes of *PARK* loci. Until now, more than 23 PD-related loci have been identified, with more being under-covered and reported [6,7], some of which are related to Mg homeostasis [8], as depicted in Figure 1. However, only 5–15% of cases belong to a group of familial forms of PD, depending on the population [9]. Numerous theories have developed over the years in an effort to explain the background behind sporadic PD pathophysiology, but none of them appear to be unifying. The review’s objective is to critically evaluate the state of the art in the field of magnesium homeostasis in relation to the risk of developing PD and suggest some potential future research directions.

## 2. Methodology

Scientific literature databases, including PubMed, Web of Science, and Scopus, were systematically queried using key terms including “magnesium homeostasis”, “Parkinson’s disease”, “animal model”, “cellular model”, “pathogenesis”, and their combinations. The presence of these terms and their combinations in the title and abstract of studies served as the main inclusion criterion. However, a study was included only if the full text in English was available. The absence of full texts and/or full texts written in another language than English served as an exclusion criterion. Studies investigating magnesium homeostasis, magnesium homeostasis disturbances, and magnesium status in animal and cellular PD models and human patients with PD were included. High-impact and recent publications were preferred in order to include up-to-date, relevant data. As the data on the role of Mg in PD pathophysiology are scarce, no temporal range filter was used during the search.

## 3. Magnesium, an “Eminence Grise” of Cellular Physiology

A highly organised system of hundreds of enzymatically catalysed chemical processes that are organised into different but interrelated metabolic and signalling pathways maintains cellular homeostasis. Numerous protein kinase-catalysed events, the Krebs cycle, lipid metabolism, carbohydrate metabolism, and nucleotide metabolism are just a few of the reactions that are thought to depend on the presence of magnesium ions (Mg^2+^). Virtually all of the cellular anabolic processes are dependent on the presence of ATP. Without Mg^2+^, this cellular energy currency is unstable in a cellular environment. Mg^2+^ controls the N-methyl-D-aspartate receptor’s (NMDAR) activity by reducing Ca^2+^ flux, which is crucial for controlling the physiology of neurons [10,11]. It has been established that aberrant NMDAR activation plays a role in the pathophysiology of several neurological and neuropsychiatric illnesses.

**Figure 2:**
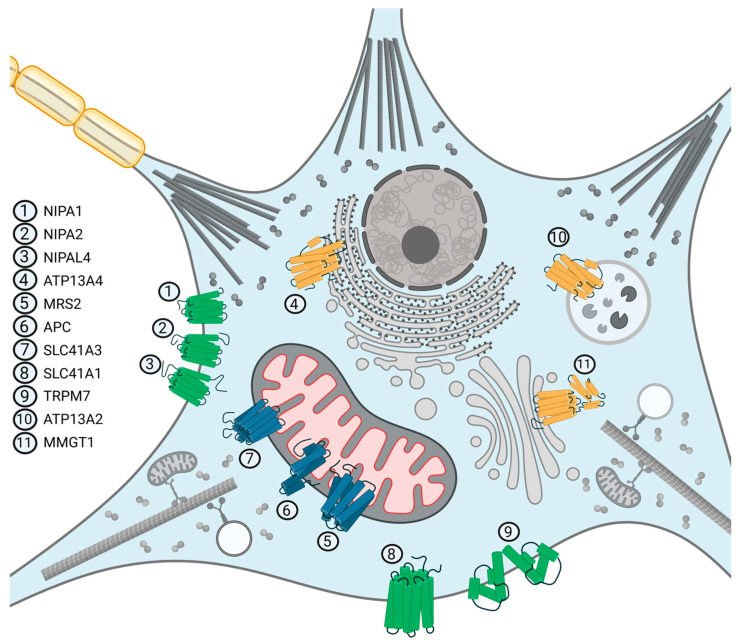
Magnesium transporters, localised in the subcellular structures of neurons. The chanzyme TRPM7 and transporters NIPA1, NIPA2, NIPAL4, and SLC41A1 belong among cell membrane proteins. ATP13A4 is localised in the endoplasmic reticulum membrane, ATP13A2 in the lysosomal membrane, and MMGT1 in the Golgi apparatus membrane. Transporters MRS2, APC, and SLC41A3 are mitochondrial membrane proteins.

Figure 2 illustrates known Mg^2+^ transporters, involved in the Mg homeostasis of neurons. More recently, it was discovered that Mg^2+^ fluxes control circadian rhythms in unicellular algae and human-derived cell lines, two types of cellular models whose phylogenetic histories split more than a billion years ago [12]. The timing of medication and potential links between shift work and the risk of chronic diseases have increased interest in the regulation of circadian clocks at the cellular and organismal levels. Additionally, several indicators point to Mg^2+^ as a potential second messenger in specific cell types [13,14]. All the aforementioned functions support the idea of Mg^2+^ being the true “eminence grise”—the behind-the-scene regulator of many key cellular functions.

In a healthy state, Mg is obtained strictly from a diet and supplements. Under disease conditions, magnesium can be prescribed as a drug, either per os or in intravenous preparation. The richest foods regarding magnesium content are seeds, nuts, legumes, cocoa, and whole-grain pastry. Magnesium-rich hard water is also a suitable and valuable source. It should be noted that around 50% of the Western population does not meet the recommended dietary intake of magnesium [15,16]. So far, magnesium has not been used in the treatment or prevention of PD, as data on its efficiency in human PD patients are scarce.

## 4. Pathomechanisms Involved in PD Onset and Progression

There are a plethora of mechanisms considered to significantly contribute to PD development on the cellular and molecular level. These mechanisms develop as a result of the long-term influence of both modifiable and nonmodifiable risk factors. The most relevant nonmodifiable risk factors are ageing, male gender, and genetic background. Modifiable risk factors, as mentioned above, include exposure to chemicals (metals, organic solvents, pesticides, and general anaesthetics), head injury, diet, and physical activity [17]. Among the most important pathological processes promoting PD onset and development are oxidative stress, mitochondrial dysfunction, neuroinflammation, and dysmicrobiosis.

### 4.1. Oxidative Stress and Mitochondrial Dysfunction in PD

Oxidative stress belongs among the most debated phenomena in a relation to PD pathogenesis [18]. Most reactive oxygen species (ROS) are produced in a process of terminal oxidation as a result of an imperfection of oxygen reduction machinery, leading to the formation of superoxide and subsequently hydroxyl radicals. Cells have developed numerous enzymatic and nonenzymatic mechanisms able to neutralise ROS. There are several crucial properties of SNPC neurons, predisposing them to be more susceptible to oxidative stress when compared to other dopaminergic neurons as well as other neuronal types in the brain. First of all, SN dopaminergic neurons require significant amounts of ATP due to their large unmyelinated axonal arbour, whose complexity and dimensions are much greater than those of other neuron classes. Propagating action potentials, maintaining the resting membrane potential, and synaptic transmission are the most prominent ATP consumers of nigral neurons [19]. Interestingly, a group of Liang [20] reported the mitochondrial mass of mouse nigral neurons to be lower compared to other neuronal types. Together, the structural and functional characteristics of nigral dopaminergic neurons necessitate considerable amounts of ATP, but these neurons also seem to have fewer mitochondria than other neuronal types. This imbalance might predispose nigral dopaminergic neurons to damage, as a minor disbalance in mitochondrial physiology could lead to the collapse of cell energy homeostasis. Such disbalance could be provoked by a significant decline in the activity of complex I of the electron transport chain as well as the activity of numerous antioxidant systems in aged nigral neurons when compared to other brain regions in healthy aged individuals, as shown by Venkateshappa and colleagues [21]. At the same time, the dysfunction of complex I accounts for the generation of substantial amounts of ROS [22]. It should be noted that complex I inhibitors (MPTP and rotenone) are currently widely used in PD research to establish PD-like pathology, as discussed in detail later. In addition to the aforementioned aspects, an increased level of ROS-mediated DNA damage, preferentially in nigral neurons, has been reported in numerous studies reviewed by Guo et al. [23]. The potential involvement of oxidative stress in PD development is supported by the fact that the use of calcium blockers [24] and serum urate levels (a potent antioxidant) [25] are associated with the risk of PD.

### 4.2. Neuroinflammation in PD

Inflammation is a complex process initiated by the immune system aiming to eliminate a cause of cell damage. It consists of a cascade of actions, starting with cytokines’ release. The production of pro- as well as anti-inflammatory cytokines is under the tight control of transcription factors, including nuclear factor κB (NF-κB), signal transducers and activators for transcription-3, the nuclear factor of activated T-cells, and activator protein-1 [26]. If the inflammatory process persists for too long, it transforms into chronic inflammation, which is detrimental. Neuroinflammation occurs in the central nervous system and represents a hallmark of multiple central nervous system (CNS) pathologies, including PD [27]. The CNS is separated from the periphery by the blood–brain barrier (BBB), which, even under physiological conditions, incompletely limits the transport of molecules and cells, relevant to the immune response of the CNS [28]. Neuroinflammation is mediated by microglia and astrocytes, which are able to respond to signals from the periphery. The putative contribution of neuroinflammation to PD pathogenesis is supported by the observation that the use of non-steroidal anti-inflammatory drugs is associated with a reduced PD risk [29]. Moreover, studies in PD model organisms suggest that the neuroinflammatory process and motor deficit might be promoted by specific gut microbiota compositions as a result of gut–brain signalling [30].

### 4.3. Autophagy and α-Synuclein Accumulation in PD

Autophagy is a process whose proper function is essential for the functioning of neurons. It is degradative machinery whose purpose is to remove redundant and/or damaged cellular components. There are three types of autophagy in mammalian cells: macroautophagy, microautophagy, and chaperone-mediated autophagy [31]. Autophagy is activated during starvation when this process recycles cellular components in order to provide nutrients essential for cell survival in a nutrient-deficient environment. Under certain circumstances, mitochondria (in a process called mitophagy), as well as other organelles and aggregated proteins, can also undergo autophagy. Several studies have shown that the processes of autophagy and mitophagy are impaired in Parkinson’s disease patients, including those with familiar forms of PD (reviewed in [32]).

A typical (and pathognomic) histological finding in the SNPC neurons of PD patients is Lewy bodies. The main components of these structures are aggregates of the fibrillar β-sheet protein α-synuclein. It is believed to be involved in the secretion of synaptic vesicles, but the exact function of α-synuclein is unknown [33]. Normally, α-synuclein is unfolded and can be degraded in several ways, including autophagy. Overexpressed as well as mutated α-synuclein interferes with the processes of autophagy [34,35]. In the case of mutant forms, chaperone-mediated autophagy and mitophagy are primarily affected. The mechanisms of autophagy and mitophagy disruption by abnormal α-synuclein are discussed in detail in the review by Hou et al. [36].

### 4.4. Dysmicrobiosis in PD

Dysmicrobiosis in PD is the result of a complex of factors, of which the most important are diet, gastrointestinal motility and genetic background. Another factor that promotes low-grade chronic inflammation is ageing. With increasing age, the immune system becomes more sensitive to external and internal stimuli. This phenomenon is referred to as a inflammageing [37]. Among theories, attempts to explain the mechanism of inflammageing are defective mitochondrial function, impaired calcium homeostasis, oxidative stress, the accumulation of somatic and mitochondrial mutations, etc. [37].

## 5. The Role of Mg^2+^ in PD Pathomechanisms

### 5.1. Magnesium and α-Synuclein

The findings regarding the influence of Mg^2+^ on α-synuclein aggregation are controversial. One in vitro study [38] analysed α-synuclein aggregation in the presence of metal cations by using atomic force microscopy. The aim of this study was to visualise and analyse the formation of spherical and annular particles composed of α-synuclein. Monomeric a-synuclein was incubated with or without metal cations (Co^2+^, Ca^2+^, Mg^2+^, Cd^2+^, Zn^2+^, Cu^2+^, Ni^2+^, and Fe^3+^) at 4 °C for a time period of one day to three months. Metal cations were added in 10-fold molar excess over protein. Incubation at 4 °C was used because of methodological obstacles in the observation of structural changes at 37 °C. The study found α-synuclein to progressively aggregate in a time-dependent manner. However, in the presence of Mg^2+^, the aggregation of α-synuclein was accelerated in terms of forming large spherical oligomeric species. On the other hand, in the presence of Ca^2+^, annular species formed rapidly. At the same time, annular oligomers nucleate filament formation. Conclusively, this study demonstrated that the oligomerization of α-synuclein was accelerated in the presence of both Mg^2+^ and Ca^2+^; however, Ca^2+^ induced the rapid formation of annular oligomers, which are precursors of a-synuclein filaments and thus more pathological.

Another in vitro study [39] analysed α-synuclein aggregation in the presence of metal cations (Fe^2+^, Fe^3+^, Cu^2+^, Mg^2+^, Zn^2+^, and Ca^2+^) at 37 °C by tyrosine fluorescence. The authors demonstrated the antagonistic effects of Fe^2+^ and Mg^2+^ on α-synuclein aggregation. Mg^2+^ was able to inhibit the aggregation of α-synuclein alone as well as in the presence of Fe^2+^. Mg^2+^ was also shown to interact with α-synuclein, but in a manner inhibiting α-synuclein aggregation. The concentration of Mg^2+^ used in the experiments was close to the physiological intracellular environment. Iron exhibited a concentration-dependent binding affinity to α-synuclein. At physiological iron concentrations, only a small amount of iron binds to α-synuclein. However, the presence of iron deposits in PD brains was reported consistently [40,41].

In a study by Hoyer et al. [42], the authors demonstrated the effect of pH and salt concentration on α-synuclein aggregates’ morphology. During the experiments, the authors used a 10 mM concentration of MgCl_2_, which led to the acceleration of α-synuclein aggregation at pH 7. However, this is a supraphysiological concentration that is far from the total magnesium concentration in serum (around 1 mM).

From the results of the aforementioned studies, it is clear that α-synuclein aggregation is highly dependent on the conditions (pH, temperature, and salt concentration) of the environment. α-synuclein is an acidic protein with pI 4.7. Lowering the pH of the environment, as well as increasing cation concentration, leads to the shielding of the negative charge of acidic residues, promoting aggregation. Regarding Mg^2+^, the results demonstrate that the effect of Mg^2+^ on a-synuclein aggregation is highly variable and depends on experimental conditions, primarily on the concentration of Mg salt and temperature. More studies are needed to clarify the effect of Mg^2+^ on α-synuclein aggregation under balanced Mg^2+^ conditions as well as in cases of Mg^2+^ deficiency in in vivo experiments.

### 5.2. Magnesium and Guamanian Amyotrophic Lateral Sclerosis and Parkinsonism–Dementia Complex

Gene coding for chanzyme TRPM7 is widely expressed among cell types, including SN neurons. This chanzyme plays a crucial role in multiple cellular processes, including Mg^2+^ transport, which is necessary for the proper function of neurons [43,44]. The zebrafish *trpm7* mutant was demonstrated to have altered the differentiation and function of dopaminergic neurons and hypomotility. These defects were partially reversed by the application of dopamine or levodopa. Moreover, mutant neurons failed to produce tyrosine hydroxylase, a key enzyme in the dopamine synthesis pathway [45]. The expression of *TRPM7* was found to be depressed in the SN of PD patients [46]. Moreover, the missense variant c.C4446T in the *TRPM7* gene was detected in a subgroup of patients suffering from Guamanian amyotrophic lateral sclerosis and the parkinsonism–dementia complex (ALS-G/PD-G). The kinase catalytic activity of the mutant protein is unaffected; however, the channel domain seems to be more sensitive to inhibition by intracellular Mg^2+^ [47]. It was debated whether the combination of Mg^2+^ and Ca^2+^ prolonged deprivation contributes to the incidence of ALS-G/PD-G with the p.T1482I variant as a higher-susceptibility factor. Cycad seed consumption is one of many theories put forth to explain the pathophysiology of ALS/PD symptoms in diverse Western Pacific communities [48]. A similar syndrome was described in cases from the Kii Peninsula in Japan, and Mg–Ca deficit was also considered as a key player here [49]. However, p.T1482I was not associated with ALS/PD in one extended Kii family [50]. Durlach et al. [51] postulated that not only a Mg deficit but a combination of a deficit of protective factors (Mg and Ca) and exposure to toxins (Al and Mn) in the diet might lead to development of the neurodegeneration. Yasui and Ota [52] supported this theory by demonstrating lower Mg content in the CNS of experimental animals fed a high-Al low-Ca diet. Purdey [53] published the hypothesis that Mg and Ca are being replaced (as a result of their deficit in diet) by ferromagnetic metals, supporting the generation of free radicals and the formation of metal–protein crystalline arrays. Together, these factors might contribute to the progression of neurodegeneration. Oyanagi’s research group [54] tried to mimic the conditions of long-term low Mg and Ca intake in two generations of rats. The authors reported a significant loss of dopaminergic neurons exclusively in substantia nigra and only in animals kept on a low Mg diet, further supporting the theory of long-term Mg deficiency as a key player in nigral neurons’ degeneration. Last, Taniguchi’s research group [55] demonstrated that a Mg deficit directly leads to the development of motor deficit symptoms in animals fed a low Mg–Ca diet.

### 5.3. Magnesium, Caffeine Intake, and Cigarette Smoking

Numerous epidemiological studies have associated cigarette smoking and caffeine intake with a reduced risk of PD development, as reviewed by Ascherio and Schwarzschild [56]. Magnesium supplementation was shown to reduce the number of cigarettes smoked by neurotic smokers as well as their Fagestrom score [57]. Even if the mechanism of protection against PD mediated by nicotinism remains unexplained, Mg deficit might serve as one of the factors promoting/keeping the habit. Caffeine was shown to decrease Mg^2+^ reabsorption in the kidney tubular system [58,59], thus contributing to Mg depletion. In general, Mg deficiency is considered to be a key player in all phases of addiction (including caffeine and nicotine) pathophysiology [60]. These findings run counter to the idea that Mg deficiency is one of the factors raising the likelihood of developing Parkinson’s disease (PD) as magnesium administration weakens one of the environmental protective variables (nicotinism) while caffeine use may cause magnesium deficiency. To better understand the function of these putative external protective factors in the development of PD, more study is required to examine the potential connections between coffee drinking, cigarette smoking, and Mg homeostasis.

### 5.4. Magnesium and Depression

Approximately 40% to 50% of PD patients develop some form of depression [61,62]. The pathophysiology of PD-related depressive disorder is multifactorial and needs to be explained in detail. The relationship between PD and depressive disorder was recently reviewed by Chikatimalla et al. [63]. There are currently two interlinked theories explaining the pathophysiological association between PD and depressive disorder: the monoamine oxidase theory and neuroinflammation. According to monoamine oxidase theory, depression evolves in PD patients as a result of the neurodegeneration of dopaminergic, serotonergic, cholinergic, and nor-adrenergic neurons. In depressed PD patients, the loss of neurons was found in the nucleus coeruleus, the primary source of brain noradrenaline, and in the nucleus raphe, a major source of brain serotonin [64,65]. The neurodegeneration of neurons is a result of an ongoing inflammation process, characterised by glial activation and pro-inflammatory cytokines’ production, as discussed in Section 4.2.

At the same time, Mg deficiency is believed to play a considerable role in the pathophysiology of major depression via multiple putative mechanisms. Serum Mg was inversely associated with depressive symptoms in a large cohort of 3604 adults [66]. Another study on 17,730 adults from the NHANES 2007–2014 cohort analysed a correlation between Mg intake and the risk of depression. The intake of Mg was assessed based on 24 h dietary recalls. The authors found an inverse, dose-dependent association between dietary Mg intake and the risk of depression development. [67]. In an open-label, blocked, randomised, cross-over trial, the authors studied the effect of oral magnesium chloride supplementation (248 mg of elemental magnesium) during a six-week period on 112 adult patients, suffering from mild-to-moderate depression. After six weeks of supplementation, there was a significant improvement in the Public Health Questionnaire—9 score (−6.0 points) as well as the Generalized Anxiety Disorders—7 score (−4.5 points). Magnesium supplementation was well tolerated; the frequency of reported side effects did not differ between groups. [68]. It is presumed that Mg works as an antidepressant in a similar modus operandi as ketamine, including the antagonism of the NMDA receptor [69], an increase in *BDNF* expression and anti-inflammatory actions [70]. Mg deficiency might thus contribute to the development of depressive symptoms in PD patients. However, data concerning the relationship between magnesium deficit and depression in PD patients are lacking. Given that depressive symptoms inflict a significant burden on the patient, who already has a major health condition, more research on this topic is extremely desirable.

### 5.5. Magnesium and Oxidative Stress

Magnesium plays a crucial role in the machinery behind DNA repair [71] and the stability of telomeres [72] and was shown to protect DNA from ROS-mediated damage when supplemented in human probands [73]. A deficiency of Mg was shown to induce oxidative damage in murine cortical neurons [74]. Mg supplementation was shown to restore antioxidant parameters in diabetic rats [75]. Kolisek et al. [76] speculated that oxidative stress-induced PARK7/DJ1 up-regulation induces Mg^2+^ efflux in cells via increasing *SLC41A1* expression. Mg^2+^ is also an essential factor in the process of ATP biogenesis on both substrate and oxidative phosphorylation levels. Moreover, as discussed later, Mg homeostasis directly affects the course of pathological processes in PD models, which are based on the induction of oxidative damage neurons. Taken together, a large body of indirect evidence suggests that perturbed Mg homeostasis may be implicated in the cellular damage brought on by oxidative damage.

### 5.6. Magnesium and Gut Microbiota

As mentioned above, disturbances in the gut microbiota might be directly involved in neuroinflammation and motor deficit development. A deficiency of Mg was shown to modify the gut microbiota in animal models, affecting the gut–brain axis [77] and provoking systemic inflammation, along with a decrease in intestinal barrier-like properties [78]. The correlation between gut microbiota composition and magnesium intake was also demonstrated in children with ADHD by Wang et al. The authors showed that *S. stercoricanis* relative abundance was increased in children with ADHD, and at the same time, Mg intake significantly correlated with *S. stercoricanis* relative abundance [79]. Magnesium supplementation was shown to rebalance the gut microbiota in a murine model of experimental colitis, favouring bacteria involved in intestinal health and metabolic homeostasis and inhibiting strains that promote inflammation [80]. The relationship between magnesium supplementation, psychiatric health, and microbiome composition was discussed in detail in a recent review by Sciopu and colleagues [81]. Improving gut microbiota composition through Mg supplementation could be a promising tool in PD prevention. However, randomised double-blinded prospective studies focused on the impact of magnesium supplementation on gut microbiota composition and disease risk and progression in patients with PD are lacking.

### 5.7. Magnesium and Sleep Disturbances

Sleep disturbances are frequent prodromal signs in many PD patients and contribute to a decreased quality of life in patients [82]. There is a solid body of evidence indicating that sleep quality is associated with magnesium status and that magnesium supplementation could affect sleep quality, especially in the elderly population [83,84]. A possible mechanism for Mg regulating sleep involves the antagonism of NMDAR [85] and agonism of gamma-aminobutyric acid receptors (GABARs) [86]. These neurotransmitter systems are involved in the sleep machinery [87]. Moreover, the levels of systemic inflammation markers are significantly increased in individuals suffering from sleep disturbances [88].

### 5.8. Magnesium and Neuroinflammation

Neuroinflammation represents one of the hallmarks of PD pathology. Mg deficiency, resulting from reduced intake, was associated with low-grade systemic inflammation. This status is accompanied by elevated levels of pro-inflammatory (CRP, TNF-α and E-selectin) cytokines and markers of inflammation that are inversely correlated with Mg intake in both healthy subjects and patients with metabolic syndrome [89,90,91]. An increased intracellular calcium level, the overactivation of NMDAR, and the inappropriate expression of NF-κB are the most debated mechanisms by which the Mg deficit putatively contributes to pro-inflammatory status [92,93]. Other mechanisms are discussed, including the overproduction of substantia P, NO, and prostaglandins. Substantia P mediates cross-talk between immune cells and neurons in terms of promoting inflammation. Mg deficiency inhibits neprilysin, the primary substantia-P-degrading enzyme [94]. As a consequence, substantia-P promotes the release of NO, ROS, prostaglandins, and cytokines from microglia [95]. Prostaglandins are produced by glial cells and neurons. These mediators of inflammation are derived from arachidonic acid and regulate neurons’ functions. The release of prostaglandin ε_2_, together with other pro-inflammatory mediators, from glial cells was shown to be attenuated by magnesium sulphate [96]. Low magnesium status affects, via promoting inflammation, cellular redox balance. Magnesium is an essential cofactor for glutathione synthesis, which is part of one of the most important cellular antioxidant systems [97].

### 5.9. Magnesium and Ageing

Oxidative stress also contributes to processes commonly referred to as ageing. Age is, at the same time, the most important risk factor for PD development [98]. Magnesium is essential for processes, determining the pace of ageing, including DNA and chromatin stability, redox balance, mitochondrial homeostasis, apoptosis, and membrane integrity [99,100]. Ageing, on the other hand, represents a risk factor for inadequate Mg (as well as other micronutrients) intake. The resulting vitious cycle of Mg deficiency promoting senescence and senescence contributing to a lower Mg intake drives clinical patterns seen during ageing. These include, among others, neurological manifestations [101].

## 6. Mg Intake and Balance in PD

The average daily magnesium intake of our Stone Age predecessors was thought to be about four times higher than that of modern Western civilisation [102,103]. Thus, it is feasible to assume that human metabolism may accommodate and also requires a higher magnesium load than the normal Western diet offers. Furthermore, a sizable amount of the Mg^2+^ content is lost during the processing of refined grains, which are a dominant part of the Western diet [104]. Another reason for the significantly reduced intake could be a decline in the Mg^2+^ concentration of agricultural soil and the limited availability of Mg for plants’ growth, resulting from soil acidity and the overuse of fertilisers [105]. It is interesting to note that the PD mortality rate was inversely correlated with soil Mg^2+^ content in 4856 samples collected from 48 states in the USA [106]. Mg^2+^ and Ca^2+^ are both well sourced from hard water. Drinking Mg^2+^-rich hard water has been linked to a lower risk of cardiovascular diseases [107], but there is no information on the risk, age of onset, or clinical course of PD in locations with access to this type of water. There is some evidence linking drinking well water to an increased risk of PD, although the findings are mixed [108]. Since there is no information on the relationship between magnesium concentration in well water and PD risk, it is expected that the presence of contamination in well water will have a greater impact on PD risk than mineral content.

### 6.1. Serum Magnesium Concentration in PD

Schwab group’s work, which found a physiological concentration of Mg in PD patients’ sera, provided the earliest information about Mg status in PD patients [109]. These early findings should be carefully considered though as the Mg measurement approach in clinical settings is, at present, far from perfect. According to a recent meta-analysis, peripheral circulating magnesium levels are in fact higher in people with PD than in healthy people [110]. Additionally, the Anirudhan group recently revealed a connection between Mg homeostasis and RPL6 expression in patients with PD [111]. The 60S ribosomal protein L6 (RPL6) was identified by the authors through meta-analysis as the core metalloprotein that connects the three hubs of the PD-related metalloprotein network. The authors observed that patient sera had greater magnesium levels than control samples in subsequent tests. At the same time, RPL6 expression and serum magnesium levels were negatively correlated. The authors postulated that PD pathogenesis might be promoted by metal homeostasis disbalance, leading to alterations of gene expression-regulating machinery. Before drawing any conclusions about circulating Mg concentration serving as a distinguishing feature of PD pathophysiology, it is necessary to take into account the heterogeneity of studies, particularly in terms of the methodologies utilised to measure Mg concentration.

### 6.2. Brain, Hair, and Cerebrospinal Fluid Magnesium Concentration in PD

Mg content was lower in PD brains than controls in the cortex, white matter, basal ganglia, and brain stem, as determined by inductively coupled plasma emission spectroscopy in 26 different brain areas [112]. Phosphor magnetic resonance spectroscopy revealed that the cytosolic Mg^2+^ content was lower in the occipital lobe of PD patients compared to controls [113]. In contrast to controls, Mg concentration was also lower in the nucleus caudatus of PD patients, according to Uitti et al. [114]. On the other hand, when Rieder et al. [115] compared clinically mild PD cases to controls, they found no variations in the regional distribution of magnesium in the postmortem brain samples.

One study indicated that PD patients had lower hair Mg concentrations [116], while other studies found that patients had noticeably greater hair Mg concentrations [117] or that there were no significant differences between PD cases and controls [118]. Interestingly, hair Mg levels were found to be higher in females in both the control and patient groups [116,118].

According to several investigations, CSF Mg levels in patients with PD were either stable [118,119,120] or influenced by clinical phenotype [121]. In 1991, Shi’s research group [122] published the results of experiments on the intracerebral autotransplantation of the adrenal medulla in 13 patients with PD. In contrast to the pre-operative state, the authors noted that patients’ clinical symptoms improved while CSF Mg concentrations remained stable. However, there was no control group, so it is impossible to conclude whether the pre-operative Mg concentration was decreased or not. Using random forest and gradient tree boosting techniques, a recent study [28] revealed Mg as a member of a group of elements (Se, Fe, As, Ni, Mg and Sr) that may distinguish between PD cases and controls with high sensitivity and specificity. Researchers discovered that patients had considerably greater levels of Mg, As, and Se in their CSF [123].

### 6.3. Magnesium Intake Assessment

As a result of a retrospective dietary questionnaire investigation on Japanese PD patients, Miyake et al. [124] revealed a potential protective benefit of greater Mg consumption along with the intake of iron and zinc. These findings conflict with a US PD interview-based patient study. The chance of developing PD was found to be higher when iron intake was higher. No association with Mg intake was found [125]. Because both of these results were acquired using either a broad questionnaire [124] or an interview [125], they should be interpreted with care. To precisely analyse the relationship between Mg intake and the risk of developing PD, more concentrated questionnaire-based research on Mg intake must be conducted. The authors Sukumar et al. recently published a validation study of a food frequency questionnaire aimed at Mg intake assessment [126], providing a valuable tool for future research.

### 6.4. Magnesium Status Assessment

Based on the results of the aforementioned studies, it is obvious that there is no definitive conclusion regarding magnesium concentrations’ differences between patients with PD and controls. Moreover, most of the studies used an interval of normal serum magnesium levels starting at around 0.75 mM. Recently, a panel of experts in a field of magnesium homeostasis proposed to increase the lower limit for hypomagnesemia to 0.85 mM [127]. The purpose of this change is to reveal cases of chronic latent magnesium deficiency. It is highly probable that if this limit was used in previous studies, a significant amount of control probands would not be suitable controls anymore, as they would be categorised as latently and subclinically magnesium-deficient. At the same time, more patients with PD would be identified as deficient regarding magnesium homeostasis. As there is virtually no consistency in reliable assessments of magnesium status, a personalised approach is essential to identify the potential deficiency of magnesium. This approach includes the determination of magnesium concentration using a strict, lower limit of 0.85 mM, detailed dietary analysis with an emphasis on the intake of magnesium-rich foods and beverages, and identification of risk factors for magnesium wasting (medication and gastro-intestinal pathologies).

## 7. Animal and Cellular Models of PD and Mg Homeostasis

Because of the nature and localization of PD pathology, it is impossible to obtain and analyse the material (SN neurons) sampled directly from patients during their lives. Studying cellular and/or animal models is currently the most widely used approach in PD pathology research [128]. However, as new techniques such as induced pluripotent stem cell generation [129] and brain organoids’ cultivation [130] emerge, it might be possible to simulate and study the PD pathomechanisms in more complex human-derived cellular and organoid models.

### 7.1. Cellular Models of PD and Magnesium Homeostasis

Numerous cellular and animal PD model-based studies have reported the significant role of Mg^2+^ in processes connected to the degeneration of neurons. Shindo et al. [131] reported protective effects of Mg^2+^ in a PC12 MPP^+^ model of PD. An increase in [Mg^2+^]_i_ was observed directly after MPP^+^ application. The authors state that the increase was provoked by the mobilisation of Mg^2+^ from mitochondria and the influx from culture medium. Intracellular Mg^2+^ levels are related to cell survival and [ATP]. The generation of reactive oxygen species was suppressed by increased [Mg^2+^]_i_. All reported effects were studied in cells cultured either in standard experimental medium containing Mg in physiological concentration or in Mg-free medium. The same group documented an increased expression level of gene encoding for SLC41A2 and a decreased expression of the genes *ACDP2 NIPA1*, and *MMGT2*. All of these genes encode for putative Mg transporter systems [132]. The modulation of the expression of these genes led to changes in the cellular MPP^+^ induced degeneration course in terms of the induction of degeneration in knock-downs and attenuation of degeneration in cases of overexpression [133].

Lin et al. [134] studied a different cellular model of PD, which was based on the treatment of cells with 6-hydroxydopamine (6-OHDA) application. The treatment of SH-SY5Y cells, a widely used cell line in PD research because of its proximity to dopaminergic neurons, with 6-OHDA led to a decrease in the cells’ viability and expression of *SLC41A1*, *NIPA1*, *MAGT1*, and *CNNM2*. *SLC41A1*, *NIPA1*, and *CNNM2* encode for systems responsible for the regulation of magnesium homeostasis [132]. Pre-treatment with MgSO_4_ avoided cell damage and reversed the decline of the aforementioned genes’ expression. These results implicate a possible protective effect of Mg^2+^ against 6-OHDA neurotoxicity. The same group [79] reported the protective effect of Mg^2+^ in a rat 6-OHDA model of PD, showing that 6-OHDA led to severe damage to dopaminergic neurons in the presence of Mg; however, the decrease in cell viability was more drastic in a group of animals that did not receive Mg. In a subsequent study, the authors detected a decrease in *SLC41A1* expression in the striatum of 6-OHDA-induced parkinsonian rats, confirming their findings from SH-SY5Y experiments [135]. The results of all the aforementioned studies indicate the crucial role of Mg^2+^ as a factor on the presence or absence of which the severity of the neurodegenerative process depends. Moreover, Mg proved to be an important player in neuronal stem cells’ differentiation and proliferation. Mg stimulation might be of great importance when it comes to neuronal progenitors’ transplantation [136].

### 7.2. Animal Models of PD and Magnesium Homeostasis

Numerous reports from animal PD models further support the role of Mg deficiency in processes associated with neurodegeneration. Lupp et al. studied the mechanism of action of memantine and amantadine (antiparkinsonic drugs) on NMDA-evoked acetylcholine release. The authors reported decreased NMDA activation in the presence of Mg^2+^ [137]. Mg^2+^ was shown to modulate the motor activity of mice treated with 1-methyl-4-phenyl-1,2,3,6-tetrahydropyridine (MPTP). Neurobehavioral studies conducted after the treatment of mice by MgSO_4_ plus MPTP and by either MgSO_4_ or MPTP alone have shown different effects of coadministration of low (2.5 g/L of drinking water) vs. medium (5 g/L of drinking water) and high (10 g/L of drinking water) doses of MgSO_4_ along with MPTP. While low doses of MgSO_4_ led to an increase in motor activity, medium and high doses led to a decrease in motor activity when compared to treatment with MgSO_4_ alone [138]. The mechanism explaining these observations is unknown; however, it is well documented that Mg^2+^ inhibits excitatory neurotransmission via NMDA. This property of Mg^2+^ seems to be beneficial and protective; however, excess Mg^2+^ might aggravate the inhibition of excitatory neurotransmission to the level of the deterioration of CNS function, which is further promoted by the presence of the neurotoxin MPTP. Furthermore, Shindo et al. [139] recently demonstrated that the exposure of rat hippocampal neurons to glutamate leads to the mobilisation of Mg^2+^ from mitochondria as a result of mitochondrial membrane potential loss. Subsequently, the Mg^2+^ exits the cell, leading to cell death. The inhibition of Mg^2+^ extrusion by amiloride and quinidine (inhibitors of *SLC41A1*—the main cellular Mg^2+^ extrusion system) improved the survival rate after glutamate treatment. Authors have also shown that the protective effect of Mg^2+^ against neurotoxicity might be mediated by mTOR signalling, in addition to the known mechanism of inhibiting of Ca^2+^ influx through NMDAR [140]. The involvement of mTOR signalling in PD pathogenesis has attracted much attention recently. However, it is still unclear whether mTOR signalling activation favours neuroprotection against neurotoxicity, as reviewed by Lan et al. [141]. Considering the main role of the mTOR complex in cell physiology—regulating cell cycle, proliferation, and metabolism—finely tuned mTOR regulation rather than up- or down-regulation might be expected to provide the best environment for the appropriate function of neurons. Mg^2+^ serves as a regulator of mTOR activity [12,142,143], which means that altered Mg^2+^ homeostasis presumably influences the activity of the mTOR complex, leading to disturbances in many aspects of cellular metabolism.

The authors Chassin et al. [144] studied the effect of the co-administration of MgSO_4_, levodopa, and benserazide (standard antiparkinsonic drugs) on the locomotor activity of MPTP-lesioned rhesus monkeys. The administration of MgSO_4_ alone did not have a significant antiparkinsonian effect; however, the co-administration of MgSO_4_ together with levodopa/benserazide exhibited a stimulatory effect on the motor responses of experimental animals. The most important observation was that Mg^2+^ significantly reduced levodopa-induced dyskinesia. This effect might have developed due to the fact that Mg^2+^ regulates metabolism and the handling of dopamine in neurons [145]. This property might be of great importance when it comes to overcoming the side effects of levodopa therapy, which are quite common among patients. Confirmatory studies in human patient cohorts are thus highly desirable.

Mg was shown to be highly protective against neurodegeneration in an MPP^+^ model of rat ventral mesencephalic–striatal co-culture [146]. MPTP administration led to a severe loss of neurons when [Mg^2+^] was 0.8 mM. However, [Mg^2+^] ranging from 1.2 mM to 4 mM abolished the toxic effect of MPP^+^ and prevented neurons’ loss and neurites’ shortening, respectively. However, Mg effectively protected neurons and neurites only when administered before MPP^+^ treatment. Mg was not able to reverse the course of neurodegeneration [146]. This observation is of importance as it provides the view that Mg is a rather potent preventive than therapeutic factor, effectively protecting neurons from ameliorating agents when present at appropriate levels. This notion significantly substantiates the necessity for adequate Mg intake in population as a prevention of developing (not only) neurodegeneration.

Mg deficiency was shown to increase the susceptibility to MPTP neurotoxicity in mice by Muroyama et al. [147]. The authors demonstrated the concomitant deteriorating effect of the Mg deficit on both the function of dopaminergic neurons and the behaviour of mice. Animals fed a Mg-deficient diet exhibited a depressive-like phenotype, as demonstrated by behavioural tests. At the same time, dopamine content in the striatum was decreased. A significant portion of patients with PD develop some form of depression, as was mentioned before. A study by Muroyama and colleagues demonstrated the development of depression behaviour in Mg^2+^-deficient animals and dopamine deficit as a result of Mg depletion under the conditions of MPTP treatment. MPTP alone was not able to decrease the level of dopamine and its metabolites in animals’ striata significantly. Taken together, the authors show that Mg deficiency might directly lead to the development of depression and that the absence of Mg aggravates the toxic effects of MPTP.

In another study, Mg-L-Threonate was shown to elevate [Mg] in CSF in a rodent model of PD. Motor deficit as well as the loss of dopaminergic neurons were attenuated when compared to MgSO_4_ treatment [148].

The Kronbauer group [149] recently demonstrated the attenuation of motor side effects in experimental animals treated with the antipsychotic drug haloperidol by Mg. Patients treated with antipsychotic drugs frequently develop motor symptoms in terms of parkinsonism and dyskinesia [150]. Mg supplementation not only prevented (and reversed) the development of catalepsy in animals but also silenced the generation of ROS by haloperidol in SN, which indirectly confirmed the findings of previously mentioned studies regarding the neuroprotective actions of Mg.

## 8. Genetic Markers in Mg-Related Genes and PD

Mg^2+^ homeostasis is directly regulated by numerous putative and confirmed transporting systems [132]. These are listed in Table 1. From the data summarised in Table 1, it is evident that most putative or confirmed Mg transporting systems are expressed in the brain, where they are involved in the regulation of Mg homeostasis in neurons on a cellular as well as subcellular level.

One of the best characterised is the Na^+^/Mg2^+^ exchanger *SLC41A1* (further referred to as NME) [151,152]. Gene *SLC41A1* was identified as a member of the *PARK16* locus, together with four other genes [153]. Subsequent research [154,155,156] confirmed this observation. Since then, multiple *SLC41A1* variants have been linked to an altered risk of PD onset and progression in both populations of Caucasian and Asian PD patients. In Han Chinese [157] and Iranian [158] populations, the synonymous variant rs11240569 was linked to a lower incidence of PD. Tucci and colleagues in British patients [159], Yaping and colleagues in Chinese patients [160], and our group in Slovak patients [161] did not, however, confirm this conclusion. Another synonymous variant, rs708727, was not associated with PD risk by Tucci et al. [159]. However, our group found a significant association between rs708727 frequency and the risk of PD [162]. It is noteworthy that this variant changed the methylation status and gene expression of the *PM20D* gene, which was linked to Alzheimer’s disease [163,164]. Multiple populations have shown an association between PD risk and the non-coding intron variant rs823156. While it was associated with a decreased risk of PD in the populations of mainland China [155], Japan [165], and Korea [166], it was not confirmed in the populations of Eastern China [167], Spain [168], Malaysia [169], Scandinavia [170], or Slovakia [161]. Bai et al. [171] performed a meta-analysis and found an inverse association between rs823156 frequency and PD risk via analysis of samples from white, Hispanic, and Asian populations, suggesting a protective role for the variant. Data from white and Hispanic populations, respectively, did not demonstrate a significant link; hence, only data from Asian populations contributed to the total significance. When interpreting the potential protective benefits of frequent single-nucleotide polymorphisms in *SLC41A1*, ethnicity appears to be significant. Kolisek et al. [172] investigated how the *SLC41A1* gene variant p.A350V (c.1049C > T) affected the way the NME functioned in HEK293 cells. The p.A350V mutation was shown to be a gain-of-function mutation since it increased Mg^2+^ efflux, as established by the authors. In addition, mutant cells showed slower development as a result of intracellular Mg^2+^ deficiency. Interestingly, the p.A350V substitution was found in one PD patient of Caucasian origin [159]. Lin’s research group [173] performed a cDNA sequencing analysis of *SLC41A1* in early-onset Taiwanese PD cases. The authors identified a heterozygous p.R244H variant in one patient with PD. The variant was found to be a loss-of-function mutation, as demonstrated in HEK293 mutant cells. Based on the evidence compiled in this section, it can be concluded that the aforementioned variants in *SLC41A1* are convincingly associated with an alteration of the risk of PD development only in certain (mostly Asian) populations. However, it is necessary to stress out that there is a plethora of putative and/or partially confirmed Mg^2+^ transporters, or Mg^2+^ homeostatic factors [132], that might be profoundly involved in PD etiopathology. Up to this point, there has not been any in-depth investigation into the presence of possible variants in genes coding those transporters or homeostatic factors in patients with PD. *SLC41A1* has been the focus of all genetic research due to its association with *PARK16*. Therefore, it is highly important to look for variations in other Mg^2+^-related genes that may be related to the development of PD. It is evident that Mg homeostasis regulation is exceedingly complex and depends on the appropriate operation of each transporting system as well as their cooperation, given the number of hypothesised and proven Mg^2+^ transporting systems. Variants in genes encoding for these systems might thus alter the expression of the gene itself or the function of a protein. In both cases, the capacity of Mg^2+^ transport might be altered, potentially resulting in intracellular and systemic deficits.

## 9. Future Perspectives

The relationship between Mg homeostasis and PD presents numerous avenues for future research. This section outlines several key areas where further investigation is needed to advance our understanding of the role of Mg in PD pathophysiology and improve patient outcomes.

**Mg and α-synuclein aggregation**: More studies are required to clarify the effect of Mg on α-synuclein aggregation under both balanced Mg conditions and Mg deficit in in vivo experiments. Understanding this relationship could provide insights into the pathological mechanisms of PD and potential therapeutic interventions.

**Mg homeostasis and lifestyle factors**: To better understand the role of external protective factors in the development of PD, additional research is necessary to examine the potential connections between coffee drinking, cigarette smoking, and Mg homeostasis. This could help identify lifestyle modifications that might mitigate the PD risk.

**Depressive symptoms in PD and Mg**: Given the significant burden depressive symptoms impose on patients with PD, further research on the interplay between Mg status and depression in this population is highly desirable. This could lead to better management strategies for depressive symptoms in PD.

**Mg supplementation and Gut microbiota**: Randomised, double-blinded, prospective studies focusing on the impact of Mg supplementation on gut microbiota composition and PD risk and progression are lacking. Addressing this gap could reveal important links between gut health and PD.

**Geographical variations in PD**: There is a need to investigate the risk, age of onset, and clinical course of PD in regions with access to Mg-rich water. This could provide epidemiological data to support or refute the protective role of Mg in PD.

**Study methodologies and Mg measurement**: Future research must account for the heterogeneity in methodologies used to measure Mg concentration. Standardising these methodologies will improve the reliability and comparability of results across studies.

**Personalised Mg status assessment**: A personalised approach is essential to identify potential Mg deficits. This includes determining Mg concentrations using a strict lower limit of 0.85 mM, conducting detailed dietary analyses, and identifying risk factors for Mg wasting, such as certain medications and gastrointestinal pathologies. To precisely analyse the relationship between Mg intake and PD risk, more concentrated questionnaire-based research on Mg consumption is necessary. These studies should aim to quantify Mg intake more accurately to assess its potential protective effects.

**Genetic variants of genes relevant for Mg transport**: An in-depth investigation into the presence of possible variants in genes coding for Mg transporters or homeostatic factors in patients with PD is lacking. Identifying these genetic factors could contribute to understanding individual differences in Mg metabolism and PD susceptibility.

Addressing these research gaps will significantly contribute to our understanding of the complex relationship between Mg and PD, potentially leading to more effective prevention and treatment strategies.

## 10. Conclusions

**Figure 3:**
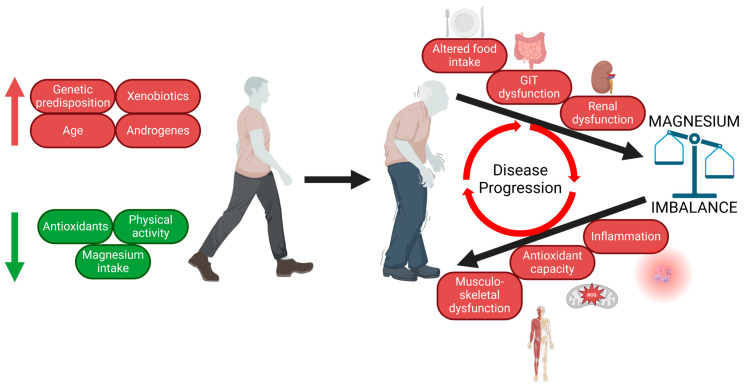
Disbalance between protective and risk factors results in PD development in senescence in most patients (sporadic PD). Higher age is associated with an increased risk of altered Mg homeostasis, which aggravates pathomechanisms and worsening clinical symptomatology. This leads to the initiation of circulus vitiosus, further contributing to the progression of PD.

A calorie-rich Western diet is poor and unbalanced regarding micronutrient content. Malnutrition affects the most vulnerable groups of the population: children and seniors. Micronutrient deficiency imposes a significant risk factor for the normal physiology of all organ systems. Perhaps the most vulnerable are the brain and nervous system. Thus, chronic malnutrition is thought to contribute to the onset of neurodegenerative diseases. PD is the most prevalent neurodegenerative disease among seniors. Altered Mg homeostasis is without doubt involved in the pathogenesis of PD (Figure 3). Research behind Mg transport and its regulation is vital for a closer understanding of the link between Mg deficiency and neurodegeneration. A personalised approach to patient care regarding Mg status assessment and subsequent Mg therapy might assist in prevention against PD and other neurodegenerative diseases.

## Figures and Tables

**Table 1:** Transporting systems involved in Mg cellular handling.

Mg^2+^ Transporting System	Mode of Action ^Δ^	Cellular Localisation ^Δ^	Detected in Brain *
NIPA1	Non-specific cation channel	Plasma membrane	+
NIPA2	Non-specific cation channel	Plasma membrane	+
NIPAL1	Non-specific cation channel	Plasma membrane	−
NIPAL4	Non-specific cation channel	Plasma membrane	+
TRPM6	Chanzyme	Plasma membrane	−
TRPM7	Chanzyme	Plasma membrane	+
SLC41A1	Na^+^/Mg^2+^ exchanger	Plasma membrane	+
SLC41A2	Putative Mg^2+^ transporter	Undetermined	+
SLC41A3	Na^+^-coupled Mg^2+^ transporter	IMM	+
MMGT1	Mg^2+^ transporter	Golgi apparatus	+
MRS2	Mg^2+^ channel	IMM	+
APC (SLC25A23, SLC25A24 SLC25A25, SLC25A41)	MgATP^2−^/HPO_4_^2−^ exchanger	IMM	+ + + +
ATP13A2	Mg^2+^ pump	Lysosomal membrane	+
ATP13A4	Mg^2+^ pump	Endoplasmic reticulum	+

* Brain localization was adapted from proteinatlas.org. “+” indicates that the protein was detected in brain and “−“ indicates that the protein was not detected in brain. ^Δ^ Cellular localizations and functions of respective transporters are adapted from [132].

## Data Availability

Not applicable.
